# The expatriate entrepreneur: Demystification and conceptualization of an international career phenomenon in the era of COVID-19

**DOI:** 10.1177/10384162221100475

**Published:** 2022-07

**Authors:** Anne-Marie Côté

**Affiliations:** 4440 Laval University, Canada

**Keywords:** Expatriation, international career phenomenon, career development, migration, entrepreneurship, expatriate entrepreneur, COVID-19 pandemic

## Abstract

International mobility brings new avenues for career development. Although the literature in human resources management has extensively investigated the traditional assignment cycle of expatriates by multinationals abroad, only few studies have focused on other forms of expatriation. Among these forms is the “expat-preneurship” whereby the expatriate decides to become an entrepreneur in the host country. This phenomenon is challenging career development in bringing new work dynamics. This conceptual paper presents a demystification of this growing phenomenon and provides a better understanding of this international career dynamic in the context of the new normal brought by the impacts of COVID-19 pandemic. Although many expatriates have opted to return home due to the fallout from the coronavirus pandemic, others have chosen to embrace an entrepreneurial career abroad. This paper sheds new light on this career phenomenon in which some individuals, despite pandemic uncertainty, see opportunities where others see roadblocks.

## Introduction

Traditionally, research on expatriation has focused on expatriates assigned by organizations for a determined term abroad (Dabic et al., 2015). However, in the current context, the nature of the international mandate and the intentions to carry it out is becoming increasingly complex. Thus, international mobility offers new roles and career paths for expatriates. We are thus seeing the emergence of a new form of expatriation, one in which individuals take charge of their own international careers without the support of an organization. These individuals, who make the decision to live and work abroad, are referred to as self-initiated expatriates (Suutari & Brewster, 2000). They resign and relocate abroad, finding employment in the host country on their own initiative (Cerdin & Selmer, 2014). These individuals can represent important resources for organizations. They generally have a good understanding of local and international markets, languages, and cultures (Al Ariss & Crowley-Henry, 2013).

Although many self-initiated expatriates find employment in local organizations or in the operations of multinationals abroad, another distinctive phenomenon seems to be gaining in importance among the expatriate community. Thus, an increasing number of expatriates are becoming entrepreneurs in the host country (Paik et al., 2017). We see the emergence of the expatriate entrepreneur or “expat-preneur” (EE) phenomenon (Solimine, 2015), which is defined as follows: “an individual temporarily living abroad who initiates a new business in the host country” (Vance et al., 2016, p. 202). However, very little research has been done on this phenomenon (Paik et al., 2017). Yet, recognition and a better understanding of this international career dynamic could have important implications, particularly for emerging countries that host EEs and organizations involved in international mobility. This paper focuses on tracing the emergence of this new phenomenon in answering the following question: What leads the expatriate to become an entrepreneur? It also extends the current discourse on expatriate entrepreneurs in exploring the phenomenon from the perspective of the new normal brought by the COVID-19 pandemic.

## Research design

The concept of EE is very sparsely addressed in the existing literature. A search in three major databases of conceptual and empirical papers in French and English published in peer-reviewed journals listed in ABDC with “expatriate” (or “expat”) AND “entrepreneur” (or “entrepreneurship”) reveals very few results. Thus, only four papers corresponding to these criteria have been identified in ABI/INFORM Global, one in Business Source Premier, and one in Web of Science.

To better understand the phenomenon, it is therefore necessary to review the existing literature as well as the key concepts that define it. As few empirical data are available (and even conceptual data), it is therefore appropriate to adopt a conceptual approach to explore and model this emerging phenomenon (Yadav, 2010). More specifically, this conceptual paper draws on a model approach that seemed the most relevant for this article (Jaakkola, 2020). The model paper contributes to extant knowledge by delineating an entity: its goal is “to detail, chart, describe, or depict an entity and its relationship to other entities” (MacInnis, 2011, p. 138). Model papers typically summarize arguments in the form of a figure that depicts the salient constructs and their relationships (Meredith, 1993).

Several concepts are involved in the definition of EE. In the first instance, we will define the foundations of the expatriation phenomenon, and then examine its links with migrant entrepreneurship before developing the conceptual framework. Finally, we will presage the effects of the COVID-19 crisis on this international career phenomenon.

## Expatriation since 1950

Research on expatriation began gradually in the 1950s. From the 1970s onward, the research field focused on multinationals and American perspectives (Dabic et al., 2015). Many of the findings of this decade were used as a basis for subsequent studies (Bonache et al., 2018). The main contributions of the studies on expatriation have been made on the concept of expatriates assigned by the organization, individuals whose careers take place within the same organization. The nature of the assignment is temporary and closely related to the tasks of the organization-sponsored job (Dabic et al., 2015).

Decades of research since the 1950s have provided relevant, albeit fragmented, results. Studies have focused on one or another of the expatriation stages: selection, preparation, international assignment, and repatriation (Dabic et al., 2015). Thus, as McNulty and Vance (2017) point out, research on expatriation continues to maintain this traditional “*circulationist perspective*,” where the expatriate's career follows a cyclical trajectory largely determined by the company's business objectives.

However, new phenomena are challenging this traditional conceptualization of expatriation (Dabic et al., 2015). International new ventures, the increase in international collaborations and self-initiated expatriates is among the realities leading to new international mobility dynamics. As organizations change, so do expatriates (Bonache et al., 2018). In this context, McNulty and Brewster (2016) present a renewed definition of the concept of expatriates: “individuals working legally and residing temporarily in a country in which they are not citizens in order to achieve professional goals, to be relocated abroad, whether through an organization or on their own initiative, or to work directly in the host country.” (McNulty and Brewster, 2016, p. 30). This definition reflects the paradigm shift in international mobility. Expatriation, long described as a strategy of American multinationals for posting executives abroad, is now tending to diversify (O’Byrne, 2018).

## The self-initiated expatriate

The literature is gradually taking an interest in a new type of expatriate: the self-initiated expatriate. Research on these expatriates began following an article by Inkson et al. (1997) on the overseas work experience of young Australians. A few years later, Suutari and Brewster (2000) introduced the concept of self-initiated expatriates, which refers to this group of expatriates, generally from developed countries, who go abroad on their own initiative to find work. Self-initiated expatriates are defined as individual who is temporarily relocated “under their own initiative” to the desired host country and gained local employment using various host country career-entry strategies (Suutari & Brewster, 2000, p. 422). The latter initiate and generally finance their own expatriation and are not transferred by any organization. One of the main objectives of self-initiated expatriates is to secure employment in the host country in order to gain international work experience and key skills (Vance & McNulty, 2014).

Self-initiated expatriates with technical and managerial skills are particularly sought after by multinationals operating in host countries and local organizations developing international markets (Tharenou, 2013). Self-initiated expatriates can also represent an alternative to assigned expatriates as they are adapted to the host country and still motivated to remain in employment beyond the end of their contract (Andresen et al., 2014). Hiring them may be less expensive for organizations since they are generally paid on a similar scale to local employees and are not entitled to the bonuses and incentives usually attributed to assigned expatriates (Dabic et al., 2015).

## The immigrant entrepreneur

Before defining what an EE is, it is first important to look at the phenomenon from a broader perspective and understand in which movement it is embedded. EEs are a very little segment of immigrant entrepreneurs. Immigrant entrepreneurs play a particularly positive role in the societies in which they operate (OECD, 2018). By pursuing careers as entrepreneurs, many immigrants successfully create income for themselves and their families, but also contribute to societies by generating novel product and service offers and employment opportunities (Kone et al., 2020).

Their growing number is one of the reasons for the ongoing scholarly interest. Upon arriving in a country, immigrants face many barriers, which would suggest entrepreneurship is a rather unlikely career choice. In comparison with natives, migrants may often lack language skills, resources, and knowledge about the market in the host country. Under those conditions, one might therefore expect that such a clear resource disadvantage against natives would lead to significantly less entrepreneurial engagement.

Evidence has suggested otherwise, however. In many countries, immigrants are as entrepreneurial as natives or are even overrepresented among entrepreneurs (Naudé et al., 2017). Self-employment is higher among the foreign-born in many developed economies, such as the United States, Canada, the United Kingdom, and Germany (Kone et al., 2020). Insights from the Global Entrepreneurship Monitor (Xavier et al., 2013) further underscore the global scale of this phenomenon: the majority of the countries surveyed report higher entrepreneurial activity among first-generation immigrants than among natives.

By the heterogeneity of their cultural and social background, immigrant and native entrepreneurs are likely to evaluate potential business opportunities differently (Madsen & Servais, 1997). However, to date, it is not clear whether and under what conditions and circumstances immigrant entrepreneurs develop different perceptions of opportunities to enter foreign markets (Bolzani & Boari, 2018). Among those immigrant entrepreneurs, however, there is a recent, as yet little-studied strain: EEs (Selmer et al., 2018) who have a singular profile.

## The EE

The concept of EE was first mentioned in an article in the *Wall Street Journal* in 2015 (Solimine, 2015). It was the subject of the first conceptual study the year after (Vance et al., 2016). A growing international career phenomenon, this segment of the expatriate population has nevertheless received scant attention compared to self-initiated expatriates.

EEs, like self-initiated expatriates, possess the classic behavioral characteristics of entrepreneurs (Vance et al., 2016). They pursue their work independently, have a high degree of self-efficacy and control, a proactive and versatile personality, and a propensity to move forward and face risk and uncertainty (Prabhu et al., 2012; Yan, 2010). They are seeking opportunities to initiate their own business development.

According to the existing research, EEs are opportunity entrepreneurs who have more flexibility and options to develop a business in the local market. Generally, from developed countries, they possess market-relevant skills that they use to observe, recognize, pursue, and exploit business opportunities in the host country (Selmer et al., 2018). This point would need to be questioned in future empirical research. Do all expatriates become entrepreneurs by choice or do some become entrepreneurs by necessity? The scant research that has been done suggests a generalization of expatriate status. It would be very relevant to explore this avenue in future research. Vance et al. (2016) distinguish two categories of EEs: the “pre-departure EE” and the “transitioned” or “post-departure EE” (2017). These two categories are described below.

## The predeparture EE

The predeparture EE (Vance et al., 2016) is the first major category of EEs. They go abroad with a clearly defined entrepreneurial intention. Predeparture EEs have plans for the development of a new business abroad even before leaving. They are clearly endowed with an entrepreneurial mindset and decide to engage in some form of entrepreneurial bricolage in the host country (Baker & Nelson, 2005). Some start a business immediately in the host country, while others will decide to repatriate the activities of an existing business to the new host country. This is not a question of setting up a head office, but of moving all the company's activities to the host country (Selmer et al., 2018).

Vance et al. (2016) distinguish another form of predeparture EE. This is the expatriate assigned by the organization taking part in the international assignment with the specific intention of looking out for business opportunities and thus leaving the multinational at the appropriate time. In this case, the traditional form of expatriation becomes a kind of instrument to obtain a placement abroad and eventually pursue entrepreneurial activities as an EE (Selmer et al., 2018; Vance et al., 2016).

## The transitioned’ or postdeparture EE

As the second major category of EEs, Vance et al. (2016) identify the transitioned EE or postdeparture EE (2017). The transitioned EE develops the ideas and intention for an entrepreneurial career only once established abroad as an assigned expatriate by an organization or self-initiated expatriate. For these transitioned expatriates, the entrepreneurial impetus is not due to a deeply rooted preexisting intention to engage in international entrepreneurship, but rather emerges from happenstance or unexpected personal or professional circumstances. Vance et al. (2016) refer to them as transitioned EEs (2016) or postdeparture EEs (2017) since their entrepreneurial career only becomes apparent to them after moving abroad. This category of EE illustrates the opportunity entrepreneur who has higher skills and is motivated by the intention to undertake while having the ability to recognize a new viable opportunity in the host country (Selmer et al., 2018).

The literature identifies another form of postdeparture EE: the traditional organization-assigned expatriate. Expatriates assigned by the organization who become EEs leave on their own initiative during or after completing their international assignment. They make the transition to new entrepreneurial activities often in the same host environment (Vance et al., 2016). This form of unplanned entrepreneurship is particularly disruptive for multinationals that invest in plans to staff and retain internationally assigned employees (Selmer et al., 2018).

## Characteristics of EEs

In a recent comparative study, Selmer et al. (2018) attempt to distinguish the characteristics of EEs from those of self-initiated expatriates. Compared to self-initiated expatriates employed by an organization, EEs are older (average age 48 vs. 43), had previously held more senior positions in organizations, had spent more time in their current job in the host country, had been expatriated for longer periods and had resided in the host country for a longer period of time. This implies that the path to becoming an EE (whether predeparture or postdeparture) is significantly influenced by temporality. Personal characteristics such as maturity and experience of living and working abroad may therefore be common traits among EEs (Selmer et al., 2018). Interestingly, education, gender, and marital status do not seem to be significant among EEs compared to assigned expatriates. These results corroborate those of Vance et al. (2017), who show that EEs have very diverse profiles.

## Conceptual framework of the EE phenomenon

An entrepreneurial career refers to a sequence of decisions and experiences regarding self-employment over the working lifespan (Dyer, 1994). The choice of entrepreneurial career may interact with other life decisions and might be revisited with the shift in personal and environmental situations. Entrepreneurial behavior is therefore influenced by a complex interplay between personal, social, and economic factors. Expatriates are particularly subject to those influences by being immersed in new environments. They are like sponge: they are driven to seek and absorb new information.

**Figure 1.**
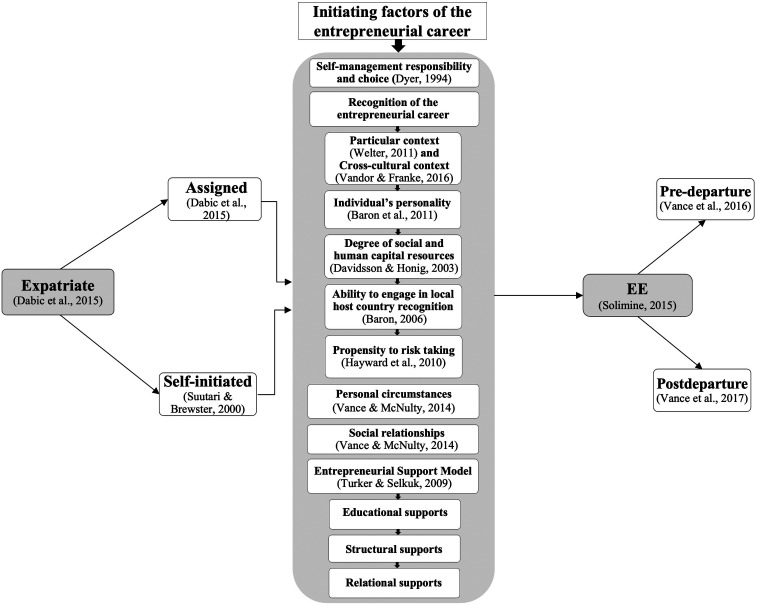
Conceptual framework of the EE phenomenon.

Various concepts and theories are mobilized in the literature in an attempt to explain what leads the expatriate to leave job stability to become an entrepreneur in the host country (Selmer et al., 2018; Vance et al., 2016). These triggers for an expatriate's entrepreneurial career are related to the themes of expatriation defined above. These linkages are schematized in the conceptual framework presented below (see Figure 1).

First of all, in accordance with Dyer’s (1994) theory of entrepreneurial career development, the EE comes to recognize his capacity for self-management and to make choices, thus disengaging from the traditional or self-initiated expatriate career in order to opt for an entrepreneurial career. According to Dyer (1994), entrepreneurs progress through various roles over the course of their careers. For the expatriate, entrepreneurship represents a career progression influenced by a series of antecedents (individual, social, and economic factors).

The recognition of the entrepreneurial opportunity by the expatriate would therefore depend on various factors. First, the context plays an important role in the recognition of entrepreneurial opportunity. According to Welter (2011, p. 165), the “economic behaviour can be better understood within its historical, temporal, institutional, spatial and social contexts.” These different contextual layers provide opportunities for individuals but also impose certain limitations on their actions. Context can be both an asset and a constraint to the nature and extent of entrepreneurship. Vandor and Franke (2016) specify a fundamental contextual layer that is important to highlight in the conceptual framework of the EE: the cross-cultural context. According to a study by Vandor and Franke (2016), “Cross-cultural experiences may increase individuals’ capabilities to identify promising business ideas.” By living in different cultures, expatriates are exposed to new products, services, customer preferences, and also way of life and thinking. This exposure may facilitate knowledge transfer and stimulate creativity. This interaction between different cultural contexts could bring new ideas, solutions and, ultimately, business models.

The individual's personality is another determining factor. As other researchers before them (Kaplan et al., 2009; Weiss & Cropanzano, 1996), Baron et al. (2011) found evidence that *dispositional positive affect* (DPA)—the stable tendency to experience positive moods and emotions—is related to several beneficial outcomes (e.g., enhanced career success, development of high-quality social relationships). To a certain extent, positive affect can be an asset to the entrepreneur and his business and can be a source of inspiration and innovation (Baron et al., 2011). Without falling into excessive positive affect, as Baron et al. point out, the individual's personality can provide an impetus to recognize the entrepreneurial opportunity.

Another factor contributing to the emergence of the entrepreneurial intention is the degree of social and human capital at his disposal (Davidsson & Honig, 2003). Having parents and friends in business and encouragement from family and friends are a powerful vector of entrepreneurial intention. According to Davidsson and Honig (2003) study of nascent entrepreneurs, identifying oneself in an established business network is also significant and positive each step of organizational emergence. These ties might be decisive in the expatriate's entrepreneurial career.

Another factor that could be a trigger for the expatriate's entrepreneurial career is the individual's ability to feel and recognize the local opportunity in the host country. It is suggested that the recognition of new business opportunities often involves pattern recognition—the cognitive process through which individuals identify meaningful patterns in complex arrays of events or trends (Baron, 2006). Research on human cognition indicates that entrepreneurs creating opportunities are able to make connections between what they already know, what they have learned in the past, and what they observe around them. This ability is rather unique and is based on the cognitive frameworks from a person's experience (Baron, 2006). New business opportunities are identified when entrepreneurs, using relevant cognitive frameworks, “connect the dots” between seemingly independent events or trends (e.g., advances in technology, shifts in markets, changes in markets, changes in government policies, etc.) and then detect patterns in these connections suggestive of new products or services. It may be assumed that expatriates are particularly endowed with this ability. As they have to adapt and integrate into their new environment, they are particularly aware of what is going on around them. They are able to perceive and create links between their previous experiences in their home country and their actual experience in the host country.

Risk-taking propensity can be defined as a person's orientation to take risks. Risk-taking propensity, which is an element of the personality of entrepreneurs, is considered to be critical for the decision to enter the entrepreneurship career (Antoncic et al., 2016). Hayward et al. (2010) argue that individuals with greater confidence can develop emotional, cognitive, social, and financial resilience. This gives them a greater ability to bounce back. The propensity to risk-taking is particularly decisive for the EE who have to accept a drop in income, at least initially. This is even more true for the assigned expatriate who have to forego a high income, generally based on the remuneration of the country of origin, and a set of compensations and benefits associated with his status (Selmer et al., 2018). The confidence in decision-making, which Hayward and his colleagues mention, thus takes on its full meaning and becomes the springboard to an entrepreneurial career.

Special personal circumstances, as well as social relations, may also be decisive in the EE's decision to stay longer in the country and start his own business. The expatriate may fall in love with the host country, its residents, or a local person. These emotional ties help strengthen the EE's social support system. They facilitate the EE's integration into local networks that promote the recognition of opportunities in the host country (Vance & McNulty, 2014).

Contextual factors can also be critical in the entrepreneurial intent of EEs. The Entrepreneurial Support Model (ESM) of Turker and Selcuk (2009) considers the impact of educational, structural, and relational supports on entrepreneurial intent. For the EE, training, and professional experience, which are elements of educational support, provide a solid basis for being able to, in the first instance, and recognize the opportunity and feasibility of an entrepreneurial project. Assigned expatriates, in particular, possess significant functional and technical knowledge and skills acquired during their assignment within organizations. This background can be very useful in the development of a new business. Not to mention their experience of organizational processes and practices acquired locally, which they will be able to transpose to support the growth and operational success of their new company (Selmer et al., 2018). The local market can also offer structural conditions that support the feasibility of the entrepreneurial project. This support from the host environment includes local and national assistance from government authorities (financing, incentives, etc.). Finally, professional relations and networks with local partners constitute the third support of the ESM model. These relational supports provide major support to EEs in starting their business. (Turker & Selcuk, 2009).

## Mediating variables

As Selmer et al. (2018) discussed in their study of expatriates, future empirical studies will need to consider certain mediating variables to have a broader understanding of the entrepreneurial intent among expatriates. Indeed, borrowing several mediating and control variables from the literature on international entrepreneurship, the expat's opportunity evaluation might be influenced by several factors: position and previous experience (Baron & Ensley, 2006), time spent abroad for any reason (travel, study, work) (Tang et al., 2012), expat's country of origin (Engelen et al., 2015), entrepreneurial family background (Bolzani et al., 2020), and expatriate's age, gender and marital status (DeTienne & Chandler, 2007). We should also consider external factors such as the firm's industry, product/service (Lorenz et al., 2018), and the local competition (percentage of competitors localized in the same region; Oviatt & McDougall, 2005). All those mediating and control variables could be tested and refined by conducting empirical studies among EEs. Additional variables that apply to the specific context of the host country could be discovered, investigated, and tested.

## The EE in the era of COVID-19

The COVID-19 pandemic has disrupted plans, changed priorities, damaged existing networks, and has led us all to question our choices. Throughout the world, many expatriates have decided to return home (Nicholas & Cadman, 2020). According to a recent survey by the estate agent Knight Frank to its global customer base, 29% of expats are considering a permanent move. The four main drivers for expatriates considering going back home are being close to family members, a new job offer, a better healthcare system, and education (Everett-Allen, 2020).

Although some expatriates have decided to return home (Rowlinson, 2020), others have seized international opportunities to start a business during the pandemic. COVID-19 has obviously inflicted severe consequences on many industries such as restaurants and bars, business and leisure travel, consulting, entertainment, and many others. Although much attention has been paid to industries experiencing challenges, other industries are booming during the pandemic. Some sectors such as technology services, home entertainment, artificial intelligence, robotics, telemedicine, medical equipment suppliers, e-commerce retailers, e-learning providers, courier pick-up and delivery services, cybersecurity, and sanitary product manufacturing have experienced growth (Zahra, 2021). These sectors represent opportunities to be seized at the international level and high-potential avenues for entrepreneurs and EEs as well.

Although some industries are growing and trends such as digital technology (e-commerce, health technology, etc.) are being strongly amplified by the pandemic (Schroeder, 2020), some countries are particularly attractive to EEs. Rather than staying in their home country, where the pandemic is hitting the economy hard and where health regulations are strict, some individuals have decided to move abroad where conditions are more favorable (Jenning & Hjelmgaard, 2020). Thus, countries with better management of the pandemic are undeniably attractive to expatriate entrepreneurs. According to a recent Bloomberg survey, which combines mortality and infection rates with COVID-19, New Zealand, Japan, Taiwan, South Korea, and Finland are among the top five countries that have best managed the pandemic.

In Taiwan, for example, a bustling technology hub of 24 million people off the coast of southeastern China, there has been no lockdown or decline in economic activity. Taiwan has registered 607 coronavirus cases in 2020 and seven related deaths, according to the Taiwan Centers for Disease Control. In this environment where the spread of COVID-19 has been relatively controlled, more than 820 residence permits for entrepreneurs were approved in 2020 compared to 358 in 2019 (Jenning & Hjelmgaard, 2020). The number of permits for foreign entrepreneurs has more than doubled over the last year.

Other countries offer favorable conditions for expatriates with entrepreneurial projects. China could potentially offer good opportunities for entrepreneurs once the immigration regulations have resumed to their normal state. A business startup visa does exist in China. It enables those having it to live in China while launching their business. As China has entered the post-COVID-19 phase, the economy is recovering more quickly. The COVID-19 pandemic has opened up a competitive landscape for startups. Their number has increased considerably surpassing the United States. With the size of the Chinese market, business opportunities are exponential. Chinese consumers have begun to act and spend largely as they did before the crisis (Sneader & Singhal, 2021). The urgent needs for professional workers, the availability of workforce at home, and a dearth of employment alternatives may have combined to create stronger momentum for entrepreneurs than in pre-COVID-19 years. Technology-spurred economic transformation seems faster and fiercer than ever in China (AsiaTechDaily, 2020).

Although some countries such as Antigua and Barbados are more inclined to receive digital nomads among which we can count EEs (Evans, 2020; Hermann & Morris Paris, 2020), some countries, including the majority of European countries, have strict entry rules and do not want nonessential workers. Whether expatriating is nonessential may be up for debate, depending on the country. In Portugal, for example, people can apply for residency visas that last four months or longer. Other countries that aren't up for casual relationships may allow people who are willing to show a little commitment, like the Dutch government, which expects entrepreneurs and self-employed workers to deposit a little more than $5,000 in the bank to demonstrate solvency (Gladstone, 2021). With travel restrictions in place and regulations frequently changing for entry/exit procedures (quarantining, COVID testing, etc.), the EE is facing many challenges through his journey. He should take into account multiple factors such as entry restrictions, health care, the nation's response to COVID-19, as well as the potential of the market in the next normal era.

## Implications

This paper presents a review of the emerging field of “expat-preneurship” that has received very little attention so far. It demonstrates that multiple personal, circumstantial, relational, and contextual factors contribute as triggers to the international career of the expatriate.

From a theoretical point of view, this paper synthesizes the literature and conceptualizes the phenomenon based on theories and themes emerging from the existing literature. It contributes to the diversification of the expatriate research field and also to the opportunity identification literature. The conceptual framework highlights different concepts and theories that can be vectors of opportunity recognition for the expatriate. These findings have clear implications for organizations, managers, entrepreneurs, and policy makers.

This paper also has important practical implications. It provides a better understanding of the issues related to the EE for both organizations and governmental authorities. As the topic is particularly sensitive for organizations assigning expatriates abroad, a better understanding of the phenomenon enables them to improve their management practices in order to offer adequate support to expatriates posted abroad, enhance their entrepreneurial behaviors and attitudes within the organization, and promote their retention. Ultimately, for government authorities, better recognition of the phenomenon may be useful in the development of effective and targeted national policies and practices to better support EEs.

## Conclusion

The recent pandemic has greatly challenged global mobility. It could be seen as a real career shock for many people across the globe (Akkermans et al., 2020). The aftermath of the COVID-19 crisis might be a key turning point—either by choice or by necessity—in many creative minds’ careers. Some experts already talk of a structural change that favors worldwide entrepreneurship, especially the technology-based kind (Brownell, 2020; Zahra, 2021). Although many in the world are still greatly impacted by the crisis, it seems obvious that there is no shortage of innovative entrepreneurs.

Despite the uncertainty brought about by the global pandemic, the phenomenon of the EE's career path seems there to stay. As we have seen in this paper, as the traditional expatriation model, expat-preneurship is subject to various mutations modeled by challenges and opportunities that arise. One of the major changes brought about by COVID-19 is the growing recognition and use of digital technology at work and in everyday life. This major trend amplified by the pandemic could benefit EEs. The growing use of digital technologies is not only leading to new business opportunities but also to the ability to work from anywhere in the world. This shift toward remote work could definitely reshape the EE phenomenon. Rather than staying in their home countries, some entrepreneurs have already taken advantage of technology to move abroad where living conditions are better.

In this paper, we use existing research to explore the evolution of expat-preneurship and presage the effects of the COVID-19 pandemic on this career phenomenon. It seems obvious that more empirical research needs to be conducted particularly in the context of the new normal. It would be relevant to validate the conceptual framework presented in this research by quantitative and qualitative approaches. Other concepts and theories (Lent et al. socio-cognitive theory of career 2002) might be applied to gain a deeper understanding of the phenomenon and its transformations. Future research attention should be paid to expatriate personal predispositions, experience, and motivations as key drivers of an entrepreneurial career. More longitudinal research is also needed to have a broader understanding of EEs’ integration dynamics and impacts on local economies. Covid effects have been extensive, and it's hard to predict its long-term consequences on global mobility and entrepreneurial career intention. However, the changes prompted by the COVID-19 crisis are likely to fuel innovation and new career opportunities worldwide.
